# Environmental Regulation, Tenure Length of Officials, and Green Innovation of Enterprises

**DOI:** 10.3390/ijerph17072284

**Published:** 2020-03-28

**Authors:** Fan Wang, Lili Feng, Jin Li, Lin Wang

**Affiliations:** 1 School of Accounting, Zhejiang Gongshang University, Hangzhou 310018, China; wangfan1031@mail.zjgsu.edu.cn; 2 School of Accounting, Hebei GEO University, Shijiazhuang 050031, China; wl642088670@gmail.com; 3 School of Management and E-Business, Key Research Institute-Modern Business Research Center, Zhejiang Gongshang University, Hangzhou 310018, China; jinli@mail.zjgsu.edu.cn

**Keywords:** Environmental regulation, tenure of officials, green innovation, environmental protection, Super-SBM data envelopment analysis (DEA), China

## Abstract

Many developing countries including China are implementing increasingly stringent environmental regulations to achieve sustainable development. However, we have limited understanding about whether environmental regulations promote enterprise green innovation. To address this research gap, this study empirically analyzes the impact of environmental regulations, which is represented by the China Environmental Protection Law (2015), on enterprise green innovation, and it explores the moderating effects of official tenure on environmental regulations and corporate green innovation. The Super-Slacks-based Measure (Super-SBM) model and multiple nonlinear regression model are employed to analyze sample data of 3557 firms in China’s A-share market during the 2014–2017 period. Our results show that, in general, a higher intensity of environmental regulations is more beneficial to incentivize enterprises to implement green innovation. Meanwhile, there is an inverted U-type relationship between the tenure length of officials and green innovation of enterprises. Furthermore, the tenure length of officials plays an inverted U-shaped role in regulating the impact of environmental regulations on enterprise green innovation. Overall, this study can help us better understand the politics behind enterprises green innovation in countries like China.

## 1. Introduction

With the potential depletion of natural resources and the aggravation of environmental pollution, in addition to economic development, environmental protection is gradually becoming an urgent task for many countries [1]. For example, in the World Environmental Performance Rankings, launched by Yale University in the United States, China’s environmental performance index (EPI) ranked 177th out of 180 countries and regions in 2018 [2]. This ranking partly reflects China’s relatively weak environmental regulations. Therefore, China is urgently trying to improve environmental regulations in time before reaching the “threshold” of ecological environment bearing; otherwise, sustainable economic growth cannot be achieved. As a result, Chinese governments at different levels formulated and issued relatively comprehensive environmental protection policies [3]. However, the problem of environmental pollution is still serious. At the same time, many developing countries like China have relatively low social welfare level, and their goals of urbanization and industrialization are not yet achieved. This means that developing countries like China also need to achieve economic growth while implementing environmental regulations [4].

“Going green” is one of the important ways that firms adapt to realize environmental protection. Various methods of acquiring green capabilities and green practices drew increased attention and discussion in the last several decades [5,6]. In particular, green innovation is critical to achieve the “win–win” goal of environmental protection and economic development [7]. Furthermore, the relationship between local government environmental regulation and enterprise green innovation was widely studied in academic research [8]. The impacts of environmental regulation on enterprise green innovation were extensively researched, but whether there is a positive “compensation effect” or a negative “offset effect” is not agreed upon. Environmental regulations will inevitably lead to increased costs for enterprises [9,10,11]. However, some scholars believe that appropriate environmental regulations can stimulate enterprise technological innovation and gain a competitive advantage [12,13]. Through environmental regulations, the government can facilitate the green development of domestic industries and create the first-mover advantage [14], and environmental taxes can accelerate technological progress and reduce environmental pollution [15,16]. The opponents, on the other hand, do not think that regulators will do any better than entrepreneurs [17]. They argue that strict environmental regulations may be detrimental to the competitiveness of enterprises [18].

Existing research attributed the inefficiency of environmental regulations to the “race-to-the- bottom” [19] behavior of local governments in the process of environmental policy formulation and implementation. If environmental quality is included as a performance measure of officials, it is bound to affect the formulation and implementation of environmental policies. However, this hypothesis is yet to be empirically tested. In addition, in many countries, local officials still hold the decision power to allocate important resources, which will directly impact the innovation strategy of enterprises. Furthermore, in general, local officials are bound in their tenure, which can play an important role in their decision-making processes [20]. On the one hand, along with a longer tenure in office, local officials can gain more expertise and experience in formulating and implementing economic policies which are conducive to enterprise green innovation. On the other hand, as the tenure of officials becomes sufficiently long, their willingness to promote green innovation may decrease, paying more attention to issues like secure retirement.

In order to fill the gap in existing studies, we take the A-share listed companies in Shanghai and Shenzhen Stock Markets as the research sample and empirically test how the intensity of environmental regulations and the tenure of officials affect enterprise green innovation. 

Our research, compared to existing studies in the literature, contributes in three major ways. Firstly, based on the Super-Slacks-based Measure (Super-SBM) model, we measure regional environmental regulations from input factors, expected output, and non-expected output. Secondly, from a macro perspective, we explore the main factors that influence enterprise innovation, environmental regulations, and the tenure length of local officials, which provides a novel way of further promoting enterprise innovation. Thirdly, we investigate the moderating effect of official tenure length on how environmental regulations impact enterprise innovation.

Overall, our study can help deepen our understanding of the important role of official tenure length in promoting environmental protection and economic development.

## 2. Background

As one of the largest developing countries and the second largest economy in the world, China adopted a development model characterized by relatively high investment, high resource consumption, and high pollution. This kind of economic model achieved remarkable economic development, but, at the same time, it has also hindered the sustainable development of the economy and society [21,22,23]. In 2012, China proposed the concept of an ecological civilization “that respects nature, conforms to nature, and protects nature, gives prominence to the construction of ecological civilization, integrates economic construction, political construction, cultural construction, and social construction into all aspects and the whole process, and strives for the sustainable development of China”. In 2015, a new environmental protection law, the strictest in history, came into force, calling for a modern environmental governance system featuring multi-dimensional co-governance and joint prevention and control. In the same year, the overall plan for the reform of the system of ecological civilization explicitly proposed the construction of the top-level design of the system of ecological civilization, including the system of performance evaluation and accountability for ecological civilization. Moreover, the officials of the local government shall be responsible for and evaluated by the local ecological civilization. In 2017, the Chinese government made an unprecedented determination to implement the “strictest system of ecological and environmental protection”. However, the situation of environmental pollution is still serious; thus, it is an inevitable choice to promote the innovation of an environmental management system where environmental regulations should be further improved.

The tenure of local officials is an important means for the central government. If the tenure is too short, officials are able to take quick and easy actions, which can lead to the opportunistic behavior of local firms. If the tenure is too long, local officials may lower their expectations of promotion and shirk their responsibilities. In China, the tenure for high-ranking local officials is five years. However, in practice, local officials are often transferred upon completing the five-year term. All in all, there seems to be no consistent tenure length for local officials [24]. Hence, in this paper, we aim to examine the impact of tenure length of local officials on firm behavior, especially firm innovation.

## 3. Theoretical Literature Review

The existing research on the impacts of environmental regulations on corporate innovation is mainly divided into two categories. The first relates to the traditional neoclassical theory. From a static perspective, it is believed that strict environmental regulations will inevitably reduce corporate profit margins, crowd out research and development (R&D) investment, and hinder corporate innovation [25]. The second category relates to the “Porter hypothesis” [12,26,27] from a dynamic perspective. It is believed that truly effective environmental regulatory policies can stimulate green technological innovation and efficiency improvements, which then accelerate innovation activities and economic growth. Furthermore, enterprises can achieve a win–win situation with high product profit and “green production”. Moreover, it was pointed out that strict environmental policies will be one of the important ways for developing countries to develop future competitive advantages [28]. In comparison, there is more research based on the “Porter hypothesis”.

## 4. Hypotheses Development

### 4.1. Environmental Regulations and Enterprise Green Innovation

The Porter hypothesis holds that environmental protection policies in the true sense will not increase the cost for enterprises. On the contrary, they can trigger innovation and generate net benefits, thus improving the international competitive advantage of enterprises [12,26,27]. On the one hand, the high intensity of environmental regulations requires enterprises to work hard to reduce pollution emissions by improving production. Enterprises usually adopt two approaches. Firstly, they control pollution level by investing in pollution control technology, which is called the “pollution control technology progress effect” of environmental regulations [29]. Secondly, they improve the productivity by improving the production technology [30]. Through technological innovation, enterprises improve the production process or improve the ability to control pollution [31]. This can eventually reduce or offset the “compliance cost” of environmental regulations and gain a competitive advantage, which is called the “innovation compensation effect” of environmental regulations. On the other hand, the government’s adoption of environmental regulations will inevitably be accompanied by certain support for technological innovation for enterprises in terms of financial policies and industrial policies. This can help solve the problem of insufficient funding for enterprise green innovation. At the same time, the implementation of environmental regulations inevitably requires the government to formulate policies conducive to environmental protection, which can incentivize enterprise green innovation. Moreover, environmental regulations can convey the signal of low resource utilization efficiency to enterprises, indicating the potential direction of technological improvement. Therefore, a reasonable environmental regulation policy can promote not only the pollution control technology, but also production technology upgrading and green innovation for enterprises. To sum up, high-intensity environmental regulations can not only incentivize enterprises to innovate and reduce pollution emission, but also result in preferential policies to reduce the capital expenses of enterprises and promote their green innovation. Based on the above analysis, the following hypothesis is proposed:

**Hypothesis** **1:** *High-intensity environmental regulations are conducive to enterprise green innovation*.

### 4.2. Tenure Length of Officials and Enterprise Green Innovation

Many researchers analyzed the government influence on enterprise innovation from the crowding out effect and the inducement effect, albeit with inconsistent conclusions [32,33]. They all generally agreed that the government is one of the important factors affecting enterprises innovation [34,35]. In particular, it was concluded that the relationship between tenure length of officials and technological innovation exhibits an inverted U-typed relationship [36,37,38].

**Figure 1:**
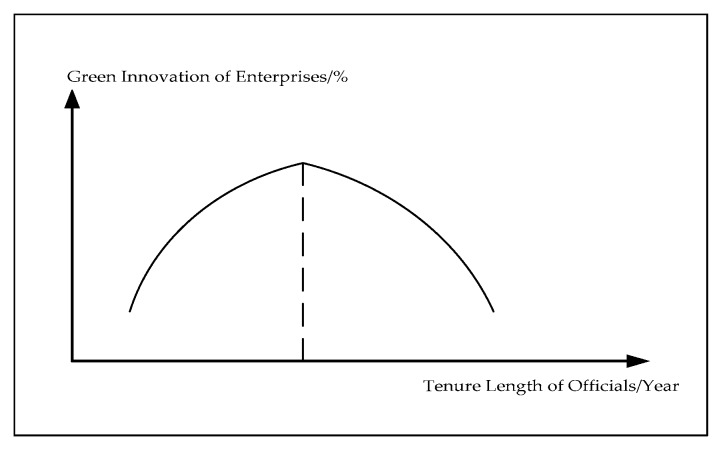
The relationship between the tenure length of officials and enterprise green innovation.

A short or transitional tenure of local officials can lead to shortsightedness and lack of focus, which is not conducive to enterprise green innovation. On the other hand, if the tenure is too long, it may lead to corruption and political association. This is also not conducive to enterprise technology innovation. In some countries, each term for critical governmental posts shall not exceed a certain number of years. In general, local officials aim for reelection, reappointment, or promotion. To this end, local officials need to achieve economic growth of their regions [39]. Hence, they will endeavor to increase investment in productive projects with quick results and low risk, while reducing investment in corporate innovation activities which may be characterized by long-term cyclicality, high uncertainty, and spillover. However, with longer tenure, local officials’ enthusiasm for short-term economic growth may reduce. They may pay more attention to the quality of economic development with more technical and forward-looking innovation. Needless to say, when local officials are approaching retirement, there is less room for their promotion. Then, what is more important for them is to how to retire safely; thus, their support for enterprise green innovation activities may decrease again. In conclusion, local officials are more likely to promote enterprise innovation at moderate tenure length. However, with further extension of their tenure, they may inhibit enterprise innovation activities, as illustrated in Figure 1. Based on the analysis above, the following hypothesis is proposed:

**Hypothesis** **2:** *There is an inverted U-shaped relationship between the tenure length of officials and the level of enterprise green innovation in their regions*.

### 4.3. Environmental Regulations and Enterprise Green Innovation: The Moderating Effect of Tenure of Officials

Local governments usually hold great discretion for many critical resources, which can give them incentives to acquire these resources through rent-seeking. However, corporate rent-seeking behavior will increase with political stability [40]. A company needs to ensure that a local official can deliver on rent-seeking commitments during their tenure. Once the official leaves office or retires, their promises may not be fulfilled, resulting in higher risk for the company. In other words, the replacement of local officials helps restrain the rent-seeking behavior of enterprises, thus affecting the implementation of environmental regulations. Therefore, the tenure of officials may moderate the relationship between environmental regulations and enterprise green innovation. When new officials take office, they may break the interest network established by their predecessors. As the new interest network is not well established, the rent-seeking behavior of enterprises will be greatly reduced. Meanwhile, under the pressure of green gross domestic product (GDP) assessment, local officials will strictly implement environmental regulatory policies to rectify local environmental pollution problems, thereby promoting corporate green innovation. However, with further extended tenure, local officials can establish their own interest network, and rent-seeking behaviors begin to occur frequently. That is, politically connected enterprises may get preferential treatment, which will reduce their “compliance cost” for environmental regulations. Based on the above analysis, the following hypothesis is proposed:

**Hypothesis** **3:** *There is an inverted U-shaped relationship where the tenure length of officials exerts mediation effects. That is, when the tenure length of officials is short, the promotion effect of environmental regulations on corporate innovation will be enhanced, but the mediation effect will gradually weaken with the extension of the tenure length of officials and even inhibit the promotion effect of environmental regulations on corporate innovation*.

## 5. Research Design

### 5.1. Data

In this paper, we selected the enterprises in China’s A-share market, including Shenzhen Stock Exchange and Shanghai Stock Exchange, as our samples. The covering period was 2014–2017. Then, according to Tian et al. (2019), we dropped those enterprises that suffered consecutive losses (marked as ST or * ST) or where serious information was missing, in order to guarantee the stability and effectiveness of our samples. After selection, we eventually obtained 3557 enterprise samples for a total of 8991 effective observations. In order to avoid the influence of extreme values, the top and bottom of related continuous variables were reduced by 1%. Related data of environmental regulations came from China Statistical Yearbook (2015–2018). The data of the tenure of officials were sorted manually as follows: firstly, we collected data on local officials from the website of the Central People’s Government of the People’s Republic of China (home page—China overview—personnel change query) and eventually obtained the names of party secretaries in 320 cities in China from 2014 to 2017. Secondly, through the database of local government officials on the People’s Network online, we found the résumés of these local government officials. Local officials with identical names were double-checked to confirm if they were the same person. Finally, the tenure of the party secretary of each city was matched manually according to their official résumé. The data on enterprise green innovation came from the Wind database, and other relevant financial indicators and corporate governance indicators came from the China Stock Market & Accounting Research (CSMAR) database. 

### 5.2. Variables

#### 5.2.1. Explained Variables: Enterprise Green Innovation

For measuring enterprise green innovation, scholars proposed a variety of methods, the most widely adopted of which is based on the ratio between research and development expenditure and operating income [41,42], denoted by Innovation (INNO). A higher INNO value denotes a higher innovation level of the enterprise, and vice versa.

#### 5.2.2. Explanatory Variables: Environmental Regulations and the Tenure of officials

(1) Environmental Regulations

In the literature, there are generally five ways to measure environmental regulations: the cost of pollution reduction [11,43]; pollution reduction [38,39]; the operating cost of pollution facilities [44] or the proportion of investment in pollution control in the total cost or output value of the enterprise; the number of industrial environmental regulations or the number of inspections and supervisions on pollution emissions by environmental regulatory agencies [45]; the ratio of GDP to energy consumption [1]. Due to the complexity of environmental policies and the different types and forms of pollutants, it is difficult to find a single index to reflect the effect of environmental regulations. Therefore, data envelopment analysis (DEA) was adopted in this study to evaluate environmental regulations.

DEA was first proposed in 1978 by American operations scientists A. Charnes, W.W. Cooper, and E. Rhodes [46]. It is a non-parametric test method developed based on the concept of relative efficiency evaluation. In DEA, the unit or organization being evaluated is called a decision-making unit (DMU). By selecting multiple input and output data of a DMU, the DEA can be categorized into five types: (1) the original Charnes-Cooper-Rhodes (CCR) model proposed in 1978 [47]; (2) the Banker-Charnes-Cooper (BCC) model which measures pure technical efficiency, that is, the ratio of technical efficiency to scale efficiency [48]; (3) the Slacks-based Measure (SEM) model which compares the efficiency among different DMUs [49]; (4) the SBM model based on relaxation variables in 2001 [50]; (5) the Super-SBM model which allows the efficiency value of effective DMU to be greater than or equal to one [51]. We employed the Super-SBM DEA model to measure environmental regulations. The Super-SBM DEA model considers undesirable outputs, which is in accordance with reality, and it is suitable for measuring the efficiency relevant to the environment. In addition, the Super-SBM DEA model overcomes the restrictions exerted by radial and piecewise linear theory. Moreover, the model can compare or sequence the decision-making units, making it feasible for input and output vectors [23,52,53].

The Super-SBM DEA model is expressed as outlined below.

Assuming there are *N* DMUs (decision-making units). Each unit contains three elements, namely, an input factor M, b_1_ expected output N^g^, and b_2_ non-expected output N^b^. We define the matrices M, N^g^, and N^b^ as follows: $M=\left[m_{1},m_{2}\cdots ,,m_{n}\right]\in {\;R}^{i\times n};{\;N}^{g}=\left[n_{1}^{g},n_{2}^{g}\cdots ,n_{n}^{g}\right]\in {\;R}^{j\times n};{\;N}^{b}=\left[n_{1}^{b},n_{2}^{b}\cdots ,n_{n}^{b}\right]\in {\;R}^{k\times n}$. We assume that M is not empty, $N^{g}\;\mathrm{is}\;\mathrm{not}\;\mathrm{empty},{\;\mathrm{and}\;\mathrm{neither}\;\mathrm{is}\;N}^{b}.$ Following Färe [54], the production possibility set (P) containing the non-expected output can be constructed as

(1) $$P=\left\{\left(m,n^{g},n^{b}\right)|m\geq M\lambda ,n^{g}\leq N^{g}\lambda ,n^{b}\geq N^{b}\lambda ,\lambda \geq 0\right\},$$

where λ$\in R^{n}$ is the intensity vector. Note that Equation (1) implies constant returns to scale. Furthermore, we can modify the SBM as follows:

(2) $$\left[SBM\right]\;\rho^{*}=\min\frac{1-\frac{1}{a}\sum_{i=1}^{a}\frac{s_{i}^{-}}{m_{i0}}}{1+\frac{1}{b_{1}+b_{2}}\sum_{j=1}^{b_{1}}\frac{S_{j}^{g}}{n_{j0}^{g}}+\sum_{j=1}^{b_{2}}\frac{S_{j}^{b}}{n_{j0}^{b}}},\;$$

$$\mathrm{subject}\;\mathrm{to}\;m_{0}=M\lambda +S^{-},\;n_{0}^{g}=N^{g}\lambda -S^{g},{\;n}_{0}^{b}=N^{b}\lambda +S^{b},$$

$$S^{-}\geq 0,{\;S}^{g}\geq 0,{\;S}^{b}\geq 0,\;\lambda \geq 0.$$

In Equation (2), the vectors S = (S^−^, S^g^, S^b^) are the relaxation variables of input factor M, expected output N^g^, and non-expected output N^b^, respectively. The value of the objective function $\rho^{*}$ represents ${\mathrm{DMU}}_{O}\left(m_{o},n^{g},{\;n}^{b}\right),$ the value of environmental regulation. If and only if $\rho_{o}^{*}=1,\;i.e.,S^{-}=0,S^{g}=0,S^{b}=0$,${\;\mathrm{DMU}}_{O}\left(m_{o},n^{g},{\;n}^{b}\right)$, is the no desired output efficient. Furthermore, the results calculated based on Equation (2) are prone to multiple DMU values of one. In order to distinguish them, the DMU with $\rho_{o}^{*}=1$ is treated as follows:

(3) $$\delta^{*}=\min\frac{\frac{1}{a}\sum_{i=1}^{a}\frac{{\bar{m}}_{i}}{m_{i0}}}{\frac{1}{b_{1}+b_{2}}\left(\sum_{j=1}^{b_{1}}\frac{{\bar{n}}_{j}^{g}}{n_{j0}^{g}}+\sum_{j=1}^{b_{2}}\frac{{\bar{n}}_{j}^{b}}{n_{j0}^{b}}\right)},$$

$$\mathrm{subject}\;\mathrm{to}\;\bar{m}\geq \sum_{j=1,\neq 0}^{t}\lambda_{j}m_{j},\;\bar{n^{g}}\geq \sum_{j=1,\neq 0}^{t}\lambda_{j}n_{j}^{g},\;\bar{n^{b}}\geq \sum_{j=1,\neq 0}^{t}\lambda_{j}n_{j}^{b},$$

$$\bar{m}\geq m_{0},\;\bar{n^{g}}\geq n_{0}^{g},\;\bar{n^{b}}\geq n_{0}^{b},\;\lambda \geq 0,$$

where $\rho^{*}$ is defined as the environmental regulation efficiency, m denotes inputs, including industrial wastewater treatment, industrial waste gas treatment, and industrial solid waste treatment, $n^{g}$ represents desirable outputs, referring to the industrial output value, $n^{b}$ is undesirable outputs, referring to those outputs that accompany desirable outputs but are not good for enterprises or do not conform to the goals of enterprises (i.e., dust, wastewater, and general solid waste), and λ is the weight vector. Related data came from China Statistical Yearbook (2015–2018). 

(2) Tenure of Officials

For measuring the tenure of officials, we used the total number of years that local officials served as the municipal party secretary. If the tenure of an official in the current year was six months or more, we counted that year; if it was less than six months, it was not counted. Particularly, if the tenure of two officials in the same year was identical, it was included in the tenure of the officials in the first half of the year. For example, official A of a city holds the post from January to June 2014, while official B holds the post from July to December of the same year as the successor. Because the government budget is usually formulated and passed in the first half of the year, and its adjustment is difficult, the year of 2014 was included in the tenure of official A.

### 5.3. Regression Model

To test the hypothesis we developed earlier, we constructed the following model:

(4) $$INNO=\beta_{0}+\beta_{1}ER+\beta_{2}Tenure+\beta_{3}Tenure^{2}+\beta_{4}ER\times Tenure+\beta_{5}ER\times Tenure^{2}\;+Control+\gamma .$$

INNO is the green innovation level of enterprise, which is measured by the ratio of research and development expenditure to operating income. Based on the Super-SBM principle of DEA mentioned above, ER was calculated according to the steps listed in Table 1 to test hypothesis H1. Tenure and Tenure^2^ respectively represent the tenure of officials and their square, in order to investigate the impact of the tenure of officials on enterprise green innovation to test hypothesis H2. It is hypothesized that the Tenure coefficient is positive and the Tenure^2^ coefficient is negative. That is, in the initial period of office, with the increase of tenure, the green innovation of enterprises increases. However, when the tenure reaches a certain peak, with the increase of tenure, the green innovation of enterprises decreases. In order to test hypothesis H3, ER × Tenure and ER × Tenure^2^, i.e., the interaction terms of environmental regulation and tenure of official, were used to investigate the impact of tenure of officials on enterprise innovation under different environmental regulations. Specifically, at the beginning of the term of official, the tenure of office enhances the enhancement effect of environmental regulation on green innovation of enterprises; however, when the tenure exceeds a certain value, the enhancement effect of the term of office weakens or even reduces. The expected coefficient of ER × Tenure is positive, and the hypothesized coefficient of ER × Tenure^2^ is negative. In addition, we followed the literature and controlled other variables that may significantly impact corporate innovation, including Size, Top1, return on equity (Roe), and debt–liability ratio (Debt). Their definitions are summarized in Table 1.

## 6. Empirical Results and Discussion

### 6.1. Descriptive Statistics

The descriptive statistics of the variables are summarized in Table 2.

**Figure 2:**
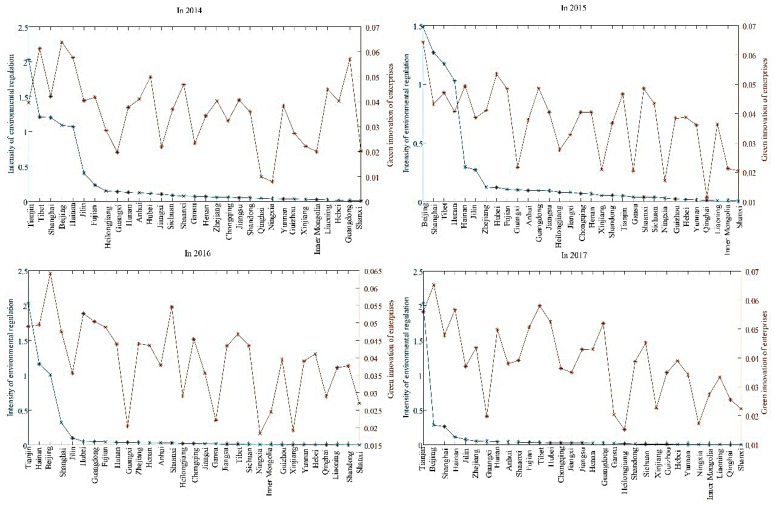
The environmental regulations and green innovation of enterprises in China in 2014–2017.

From Table 2, we can see that the average level of enterprise green innovation (INNO) is 0.045, which indicates that the innovation level of the samples is generally low, and the government may need to take measures to incentivize enterprises to actively carry out innovation activities. In terms of explanatory variables, the mean value of environmental regulations (ER) is 0.21, the maximum value is 2.03, and the minimum value is 0.001. This indicates that there are pronounced differences in the intensity of environmental regulations in various regions. The maximum value of tenure (Tenure) is 13, and the minimum value is one, indicating that there is a gap in tenure of officials. Its mean value is 4.36, indicating that the tenure of local municipal party secretaries is about five years. Moreover, the environmental regulations and green innovation of enterprises in 31 provinces and regions in 2014-2017are illustrated in Figure 2.

From Figure 2, it can be seen that, since the implementation of the new environmental protection law, environmental regulations and green innovation of enterprises in various provinces and regions changed, especially between 2016 and 2017. The level of enterprise green innovation is high in provinces and regions with high environmental regulation, and vice versa. In addition, the regional differences in environmental regulation are rather significant. Except for the top five provinces and regions, such as Tianjin, Beijing, and Shanghai, the efficiency of environmental regulation in other provinces is generally low.

In terms of control variables, the average enterprise size is 22.08, the maximum value is 26.11, and the minimum value is 19.91, indicating that there is a large difference in enterprise size. The average value of return on equity (Roe) is 0.07, the maximum value is 0.30, and the minimum value is −0.42, indicating that the profitability of listed companies in the sample is generally low. The average shareholding ratio of the largest shareholder (Top1) is 34.33%, the maximum is 89.09%, and the minimum is 3.39%. It can be concluded that the shareholding ratio of the largest shareholder of an enterprise is relatively scattered. The average value of debt ratio (Debt) is 0.40, which means that most enterprises will promote their development with a leverage effect. In the process of calculating the descriptive statistics, some variables were found to have extreme values, which will be dealt with in subsequent empirical research.

### 6.2. Correlation Test

The correlation test among variables was carried out, and the results are summarized in Table 3.

From Table 3, we can see a significant positive correlation between environmental regulation (ER) and enterprise green innovation (INNO) at the level of 1%, which preliminarily supports hypothesis H1; there is a significant positive correlation between official tenure (Tenure) and enterprise green innovation at the level of 1%. In addition, there is a significant negative correlation between enterprise size (Size) and enterprise green innovation at the level of 1%, which means that the smaller listed companies pay more attention to green innovation, possibly due to the greater survival pressure. The relationship between Roe and INNO needs further exploration. The shareholding ratio of the largest shareholder (Top1) is significantly negatively correlated with the green innovation level at the level of 1%, indicating that shareholding dispersion is not conducive to green innovation activities of enterprises. Moreover, debt is negatively correlated with INNO at the level of 1%. In brief, those correlation test results show that environmental regulations and tenure of officials can affect the level of enterprise green innovation to a certain extent. In addition, the correlation coefficients among the explanatory variables are sufficiently low, which indicates no serious multicollinearity problem in the model.

Table 4 presents the variance inflation factor (VIF) analysis for each variable. All VIF values were less than 10, which suggest that multicollinearity is not a concern.

### 6.3. Regression Analysis

In order to test the hypothesis developed in this paper, we conducted multiple regression based on Equation (4). The results are summarized in Table 5.

Table 5 lists the regression results on the relationships among environmental regulations, tenure length of officials, and enterprise green innovation. Column 1 shows a significant positive correlation between environmental regulations and enterprise green innovation at a significance level of *p* < 0.01. In other words, higher environmental regulations make it more conducive for enterprise green innovation. Therefore, hypothesis H1 is supported, which is consistent with the conclusions in the literature [59,60,61] In column 2, the regression coefficient of enterprise green innovation to the first term of tenure of is 0.001 at a significance level of *p* < 0.01, while the coefficient of the square term is significantly negative at a significance level of *p* < 0.01. Therefore, hypothesis H2 is supported. This is consistent with Reference [62]. Hence, there is an inverted U-shaped relationship between tenure length of officials and enterprise green innovation. That is, as the tenure length of officials continues to increase, the level of enterprise green innovation firstly increases and then decreases. Furthermore, our analysis reveals that the inflection point in the inverted U-shaped curve is about five years. This is a new finding. Interestingly, the average tenure of the local officials in our sample is about 4.36 years, which is less than the inflection point of the curve. This indicates that the impact of the tenure of officials on enterprise green innovation is in the upward part of the inverted U-shaped curve. In column 3, the interactive term between environmental regulations and tenure (ER × Tenure) is significantly positively correlated with enterprise green innovation at a significance level of *p* < 0.01. However, the interaction term between environmental regulations and the tenure (ER × Tenure^2^) is significantly negative at a significance level of *p* < 0.05. Thus, hypothesis H3 is also supported. This implies that tenure strengthens the influence of environmental regulations on enterprise innovation in the beginning. However, with increasingly longer tenure, this positive reinforcement can gradually weaken or even become negative. This may be caused by the fact that officials with short tenure are eager to highlight their achievements. Hence, they pay more attention to short-term economic growth, which is not conducive to enterprise green innovation. With longer tenure, local officials can develop richer social capital and focus on long-term economic growth through enterprise green innovation. However, if the tenure is too long, officials may be able to establish a solid social network of interest, where enterprises can obtain preferential treatment and reduce the “compliance cost” of environmental regulation policies. In this way, it can weaken or even hinder enterprise green innovation. This implies that the central government should fully consider its impact on enterprise behavior when formulating the tenure system of local officials, and it should maintain consistent policies as much as possible.

In addition, in terms of the control variables selected in column 1 to column 3, the company size (Size) and the shareholding ratio of the largest shareholder (Top1) are significantly negatively correlated with the level of enterprise green innovation at a significance level of *p* < 0.01, indicating that a larger scale and more dispersed equity are more detrimental to enterprise green innovation. Moreover, return on equity (Roe) is significantly negatively correlated with enterprise green innovation at a significance level of *p* < 0.01. In addition, the debt ratio (Debt) is significantly negatively related to the level of enterprise green innovation at a significance level of *p* < 0.01, which may be due to the fact that, with a higher debt ratio, indicating a greater pressure of debt repayment, an enterprise is less likely to carry out innovation activities.

### 6.4. Robustness Check

In this paper, we chose the DEA value calculated from Equation (2) as an alternative variable of environmental regulations and conducted the regression tests again. The results are summarized in Table 6. Based on Table 6, we can see that the regression results did not change substantially. The coefficients of the core explanatory variables are in accordance with the theoretical expectations. Furthermore, there are good levels of statistical significance, which means that the empirical results of this study are robust. 

## 7. Summary and Conclusions 

In this paper, we assessed the impact of environmental regulations and tenure of officials on enterprise green innovation. To this end, we analyzed 3557 enterprises in the A-share market for the 2014–2017 period. The Super-SBM DEA model was employed to explore environmental regulation efficiency. We examined whether environmental regulations and the tenure of officials had a negative or positive impact on enterprise green innovation. Furthermore, we also conducted robustness analyses and heterogeneity tests for our results.

We found that, in general, high-intensity environmental regulations positively influence enterprise green innovation. More specifically, there is a significant inverse U-type relationship between the tenure length of officials and enterprise green innovation. That is, as the tenure of officials increases, the level of enterprise green innovation will firstly increase until a threshold, after which it will decrease. In addition, we found that maintaining a tenure length of about five years seems the optimal level to promote green innovation. More importantly, there is an inverted U-shaped relationship when the tenure length of officials exerts mediation effects. In other words, when the tenure length of officials is short, the promotional effect of environmental regulations on corporate innovation will be enhanced. However, the mediation effect will gradually weaken when the tenure of local officials is further extended. In summary, this study can help us better understand the politics behind enterprise green innovation in countries like China. 

Our empirical results also led to two policy insights. Firstly, the government can further improve environmental regulations to fully unleash the positive effects of environmental regulations on enterprise green innovation. For example, local officials can formulate financial policies to support enterprise technological innovation. Secondly, the government should set an appropriate tenure length for local officials considering the issues of environmental protection and green economy development. At the same time, the tenure system of local officials should ensure the continuity of related policies on environmental protection and green economy development. In addition, different performance appraisal and incentive policies may be required for local officials with different tenure length. This research can be extended in several ways. Firstly, a limitation of this study was our specific measurements of several variables. For example, although content analysis was used to measure environmental regulations, it may be unable to capture the actual achievement due to environmental regulations. Hence, in the future, it is necessary to further improve the measurement of environmental regulations. Secondly, our data were limited to the publicly listed companies in China. It would be interesting to examine our research in a multi-country context. Last but not the least, future research can further focus on how environmental regulations impact a specific green innovation activity like green product design [63]. Further research can also focus on analyzing the relationship between the government and enterprise innovation using analytic models [64].

## Figures and Tables

**Table 1:** Variable definitions. R&D—research and development

Variable Types	Variables	Definitions and Measurements	Literature
Explained variable	INNO	R&D expenditure/revenue	[41,42]
Explanatory variables	ER	(1) Environmental regulation (ER) index, i.e., the calculation method in Section 5.2.2.(1); (2) Ranking the DEA value in order from large to small; (3) Taking the reciprocal of the order; (4) Returning the reciprocal as the valve of environmental regulation.	[55]
	Tenure	The tenure of an official calculated as described in Section 5.2.2.(2)	
	Tenure^2^	The square of the number of years an official held office	
	ER × Tenure	Interaction between environmental regulation and tenure of officials	
	ER × Tenure^2^	Square interaction term between environmental regulation and the tenure of officials	
Control variables	Size	Total assets at the end of the natural logarithm	[4,23,56]
	Top1	The shareholding ratio of the largest shareholder	[57]
	Roe	Net profit/average net assets	[4,58]
	Debt	Total liabilities at end/total assets at end	[57,58]

**Table 2:** Descriptive statistics of variables.

Variables	Observations	Mean	SD	Min	Max
INNO	8991	0.0448	0.0436	0.0030	0.2544
ER	8991	0.2121	0.4116	0.0010	2.0331
Tenure	8991	4.3625	3.0138	1.0000	13.0000
Size	8991	22.0767	1.2704	19.9129	26.1053
Roe	8991	0.0666	0.0955	−0.4223	0.3029
Top1	8991	34.3329	14.7411	3.39	89.09
Debt	8991	0.3966	0.1981	0.0552	0.8719

**Table 3:** Correlation coefficients of main variables.

Var	INNO	ER	Tenure	Size	Roe	Top1	Debt
INNO	1.0000	0.0616 ***	0.0853 ***	−0.3596 ***	0.0308 ***	−0.1649 ***	−0.3605 ***
ER	0.0932 ***	1.0000	0.1943 ***	−0.0106	0.0054 *	0.0282 ***	−0.0150
Tenure	0.1047 ***	0.1947 ***	1.0000	0.0296 ***	0.0036	0.0119	0.0110
Size	−0.2824 ***	0.0614 ***	0.0418 ***	1.0000	0.0124	0.1012 ***	0.5562 ***
Roe	−0.0249 **	0.0039	−0.0056	0.0190 *	1.0000	0.1391 ***	−0.1035 ***
Top1	−0.1681 ***	0.0466 ***	0.0112	0.1574 **	0.1063 ***	1.0000	0.0684 ***
Debt	−0.3163 ***	−0.0055	0.0104	0.5656 ***	−0.1741 **	0.0782 ***	1.0000

Note: the upper right part of the table shows the Spearman test, and the lower left part shows the Pearson test. ***, **, and * respectively represent statistical significance levels of 1%, 5%, and 10%.

**Table 4:** Variance inflation factor (VIF) statistics. Mean VIF: 1.21.

Variable	VIF	ER
ER	1.05	0.955822
Tenure	1.04	0.961085
Size	1.53	0.652039
Roe	1.07	0.938797
Top1	1.04	0.963473
Debt	1.55	0.644229

**Table 5:** Regression coefficients.

Variable	(1)	(2)	(3)
ER	0.0002 *** (4.107)		
Tenure		0.001 *** (6.565)	
Tenure^2^		−0.0001 *** (−2.547)	
ER × Tenure			0.00005 *** (7.296)
ER × Tenure^2^			3.82 × 10^−6^ ** (−2.060)
Size	−0.001 *** (−3.781)	−0.002 *** (−4.100)	−0.002 *** (−4.303)
Roe	−0.034 *** (−8.393)	−0.033 *** (−8.142)	−0.033 *** (−8.314)
Top1	−0.0002 *** (-6.264)	−0.0002 *** (−6.270)	−0.0002 *** (−6.499)
Debt	−0.046 *** (-19.481)	−0.046 *** (−19.636)	−0.046 *** (−19.406)
Ind/Year	Yes	Yes	Yes
Constant	0.092 *** (2.631)	0.085 *** (2.414)	0.089 ** (2.538)
Observations	8991	8991	8991
F	68.99	68.79	69.10
R-squared	0.3971	0.3992	0.4003

Note: The standard error of each estimated value is provided in brackets; *** represents *p* < 0.001, ** represents *p* < 0.05.

**Table 6:** Regression coefficients for the robustness check model.

Variable	(1)	(2)	(3)
ER	0.005 *** (5.306)		
Tenure		0.001 *** (6.565)	
Tenure^2^		−0.0001 *** (−2.547)	
ER × Tenure			0.001 *** (2.971)
ER × Tenure^2^			0.0003 (1.319)
Size	−0.002 *** (−4.107)	−0.002 *** (−4.100)	−0.002 *** (−4.350)
Roe	−0.033 *** (−8.282)	−0.033 *** (−8.142)	−0.033 *** (−8.184)
Top1	−0.0002 *** (−6.355)	−0.0002 *** (−6.270)	−0.0002 *** (−6.447)
Debt	−0.046 *** (−19.423)	−0.046 *** (−19.636)	−0.046 *** (-19.334)
Ind/Year	Yes	Yes	Yes
Constant	0.091 *** (2.597)	0.085 *** (2.414)	0.093 *** (2.668)
Observations	8991	8991	8991
F	69.21	68.79	68.83
R-squared	0.3978	0.3992	0.3993

Note: The standard error of each estimated value is provided in brackets; *** represents *p* < 0.001.
